# Supplementary material for: Rapid and robust restoration of breathing long after spinal cord injury
Source: Nat Commun. 2018 Nov 27;9:4843. doi: 10.1038/s41467-018-06937-0 (PMC6258702; doi:10.1038/s41467-018-06937-0)
Supplement: Supplementary file 1 — Supplementary Information [file 41467_2018_6937_MOESM1_ESM.pdf]

Supplementary Information

Title: Rapid and robust restoration of breathing long after spinal cord injury

Warren *et al.*

Supplementary Materials:

Supplementary Figures 1-10

Supplementary Tables 1-3

Supplementary figures:

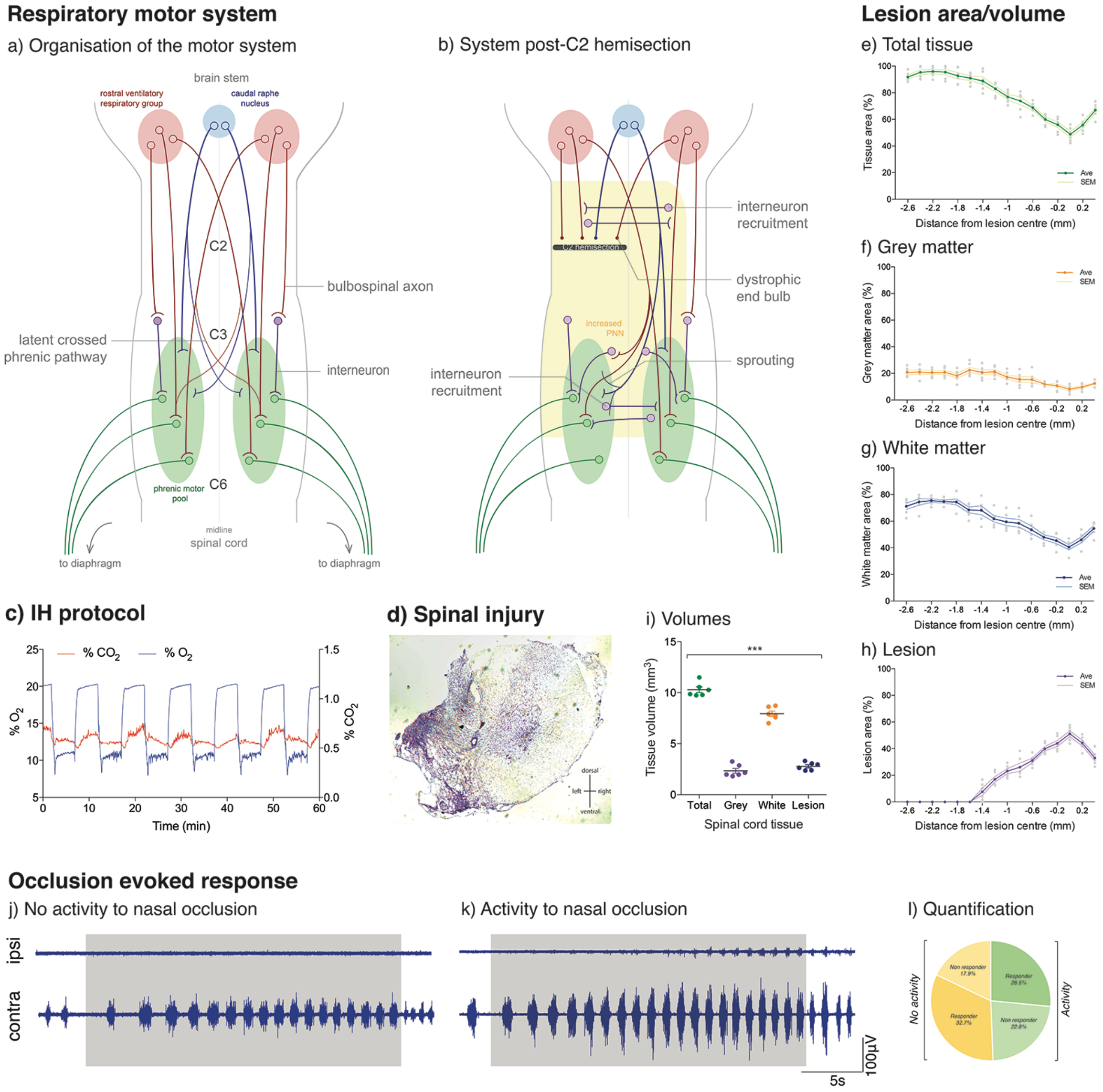

Supplementary Figure 1. Injury model and treatment paradigm. a-b) Schematic of descending inputs to inspiratory respiratory motor function of the diaphragm both a) before and b) after a lateral C2 hemisection. c) Representative trace of the IH protocol. Training took place five days a week, comprising of 10 hypoxic five min episodes followed by five min rest periods in air (normoxia). Data shows % O_2 (blue line) and CO_2 (red line) over time. Note, the concentration of CO_2 did not accumulate, thus any effect of conditioning is caused by IH alone. d) Representative spinal cord C2 hemisection injury stained with crestyl violet. e-i) Percentage area of e) spinal tissue (green, ±SEM = light green), f) grey matter (orange, ±SEM = yellow), g) white matter (blue, ±SEM = light blue), h) the lesion (purple, ±SEM = light purple), over distance from the injury site and i) volumes of selected tissue (total tissue = green, grey matter = orange, white matter = blue; n = 6). j-l) Illustration of the occlusion evoked response showing j) no activity, k) activity from the ipsilateral hemidiaphragm and l) quantification (no activity = yellow, activity = green). The grey box demonstrates the time of occlusion. Panels show means ± SEM with line graphs additionally showing individual data points in grey. *** = $p<0.001$ (*Bonferroni*).

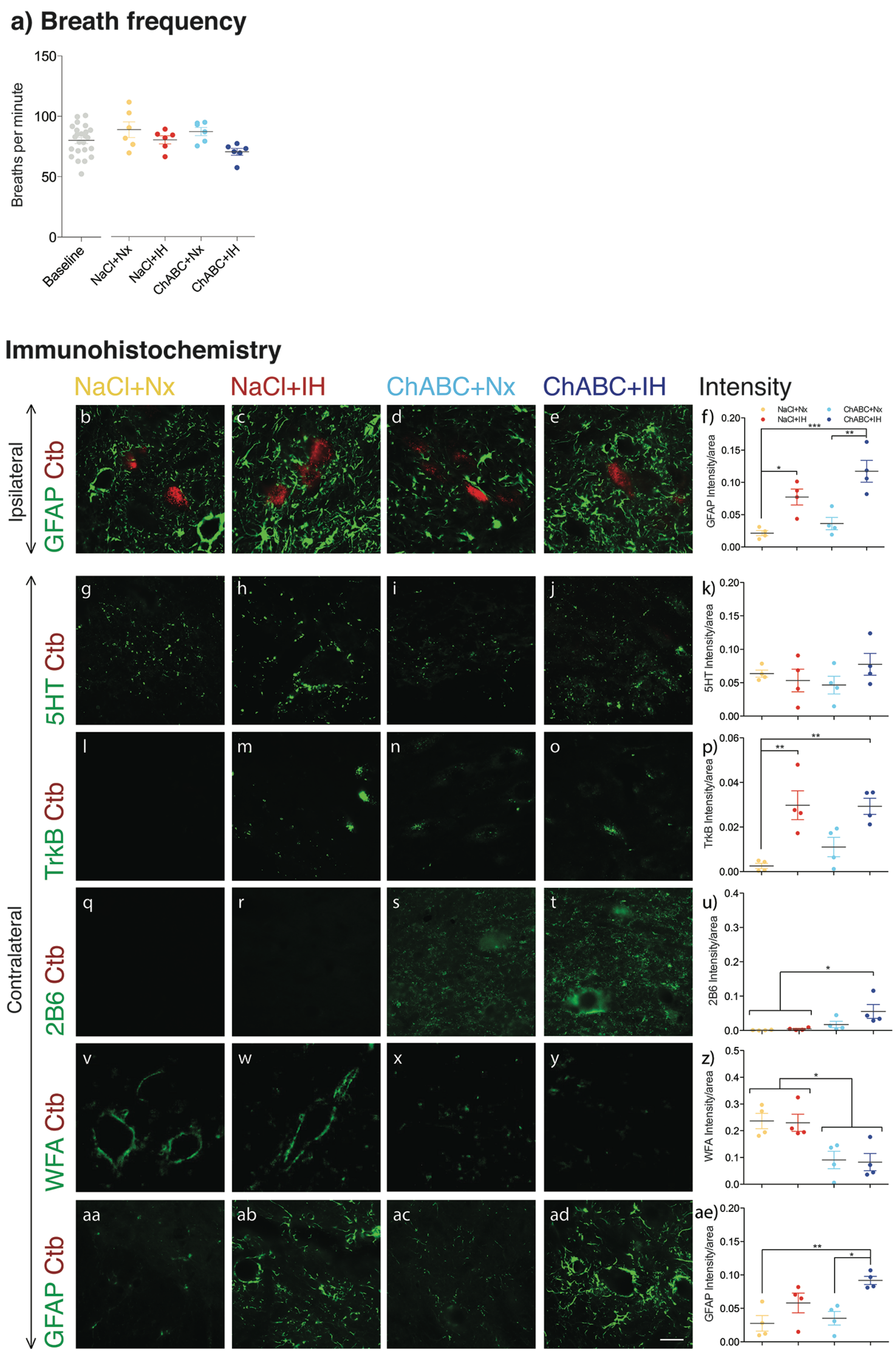

Supplementary Figure 2. PNN decreases bilaterally following ChABC treatment in twelve week chronically injured animals. a) Average number of breaths per minute for each treatment group (n=6 per group, baseline n=24). B-ae) Immunohistochemistry and intensity readings at the b-f) ipsilateral and g-ae) contralateral C4 PMP for all treatment groups utilizing Ctb tracer (red) and either (green) b-f and aa-ae) GFAP, g-k) 5HT, l-p) TrkB, q-u) 2B6, or v-z) WFA. For all graph panels treatment groups = baseline (grey); saline+air (yellow); saline+IH (red); ChABC+air (light blue); ChABC+IH (blue). * = $p<0.05$, ** = $p<0.01$, and *** = $p<0.001$ (*Bonferroni*) (n=4 per group). If no post-hoc result is shown, comparison was not-significant. Scale bar = 50µm. For all panels: values represent mean±SEM.

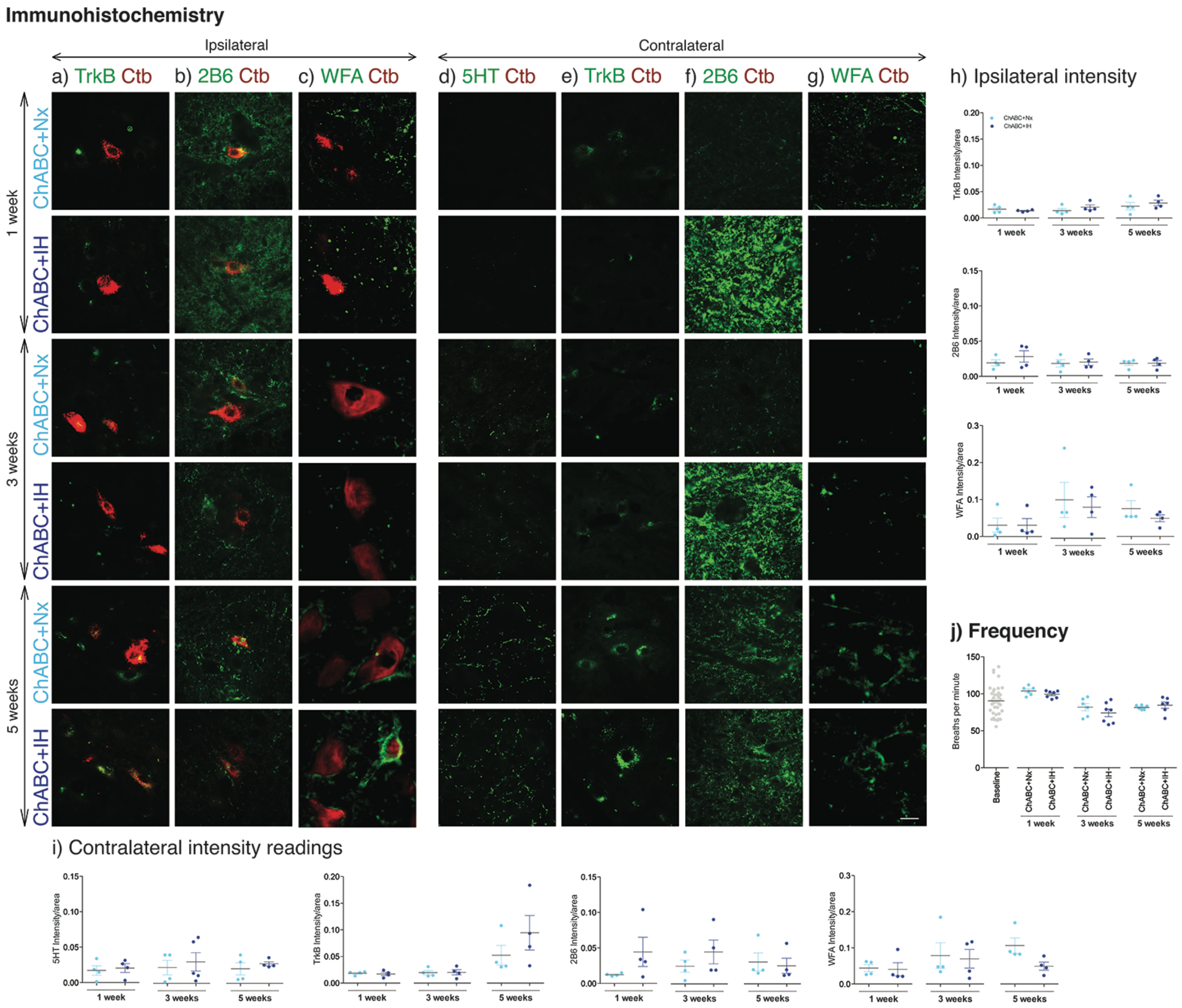

Supplementary Figure 3. PNN is slowly re-established following injection of ChABC twelve weeks after C2 hemisection. a-i) Immunohistochemistry and intensity readings at the a-c) ipsilateral and d-g) contralateral C4 PMP for all treatment groups utilizing Ctb tracer (red) and either (green) a and e) TrkB, b and f) 2B6, c and g) WFA, or d) 5HT. Scale bar = 50µm (n=4/5 per group). (J) Average number of breaths per minute for each treatment group (n=6/7 per group, baseline n=38). For all graph panels treatment groups = ChABC+air (light blue); ChABC+IH (blue). * = $p<0.05$ (*Bonferroni*). If no post-hoc result is shown, comparison was not-significant. For all panels: values represent mean±SEM.

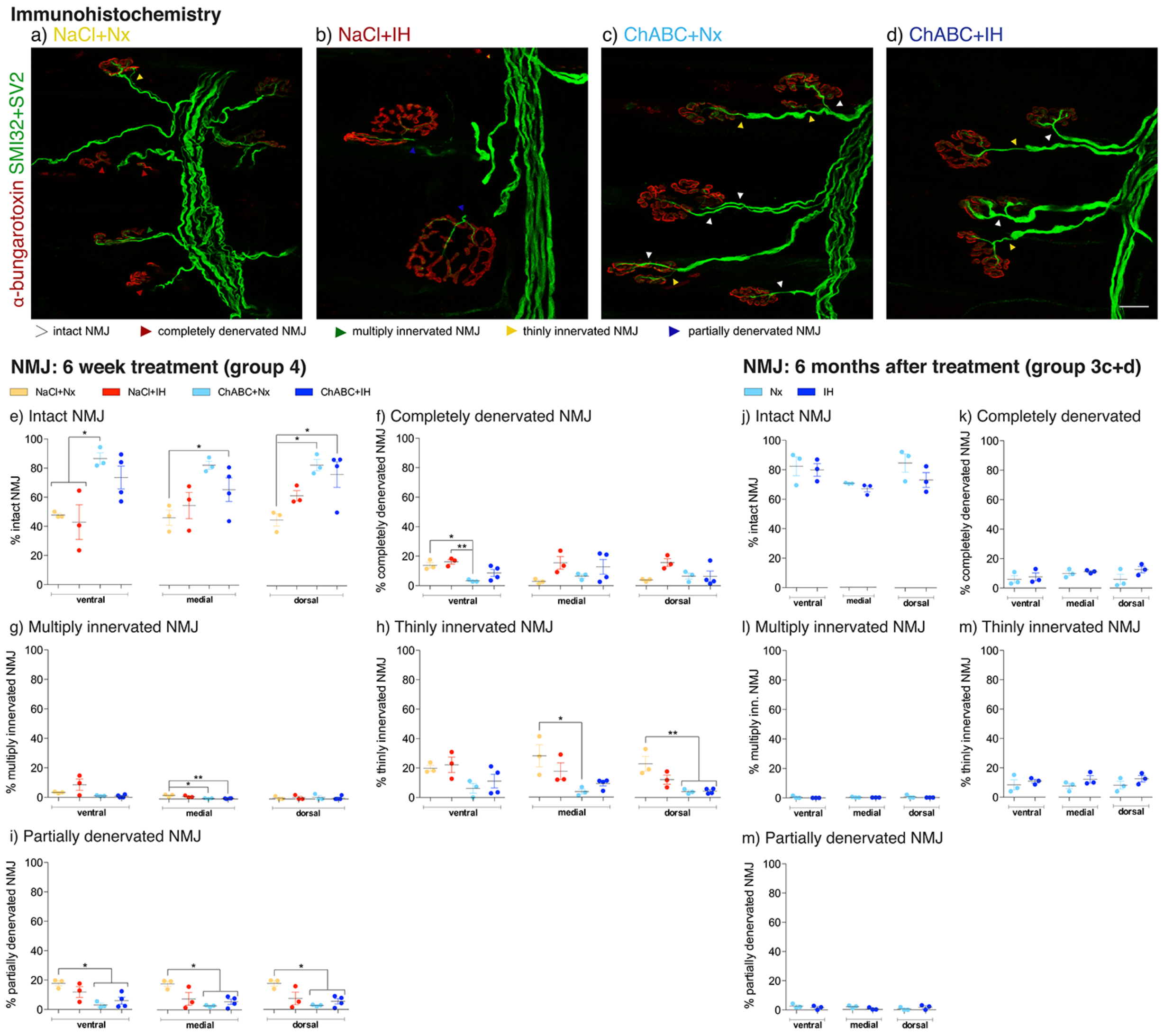

Supplementary Figure 4. PNN degradation facilitates increases in intact ipsilateral neuromuscular junctions (NMJs). a-d) Animals were injured for twelve weeks and received ChABC or NaCl and 5 weeks of IH conditioning or air. NMJs labelled with rhodamine-alpha-bungarotoxin (red), SMI-312R (green) and SV2-s (green). For all triangles on image panels: white = intact NMJs; red = completely denervated NMJs; green = multiply innervated NMJs; yellow = thinly innervated NMJs; blue = partially innervated NMJs. e-i) Quantification of the ipsilateral hemidiaphragm NMJ analysis. j-m) Quantification of ipsilateral hemidiaphragm NMJ six months after the end of treatment. Animals were injured for twelve weeks prior to ChABC, and received only one week of conditioning before being housed for six months. For all graph panels treatment groups = saline+air (yellow); saline+IH (red); ChABC+air (light blue); ChABC+IH (blue). * = $p<0.05$, and ** = $p<0.01$(*Bonferroni*) (n=3/4 per group). If no post-hoc result is shown, comparison was not-significant. For all panels: values represent mean±SEM.

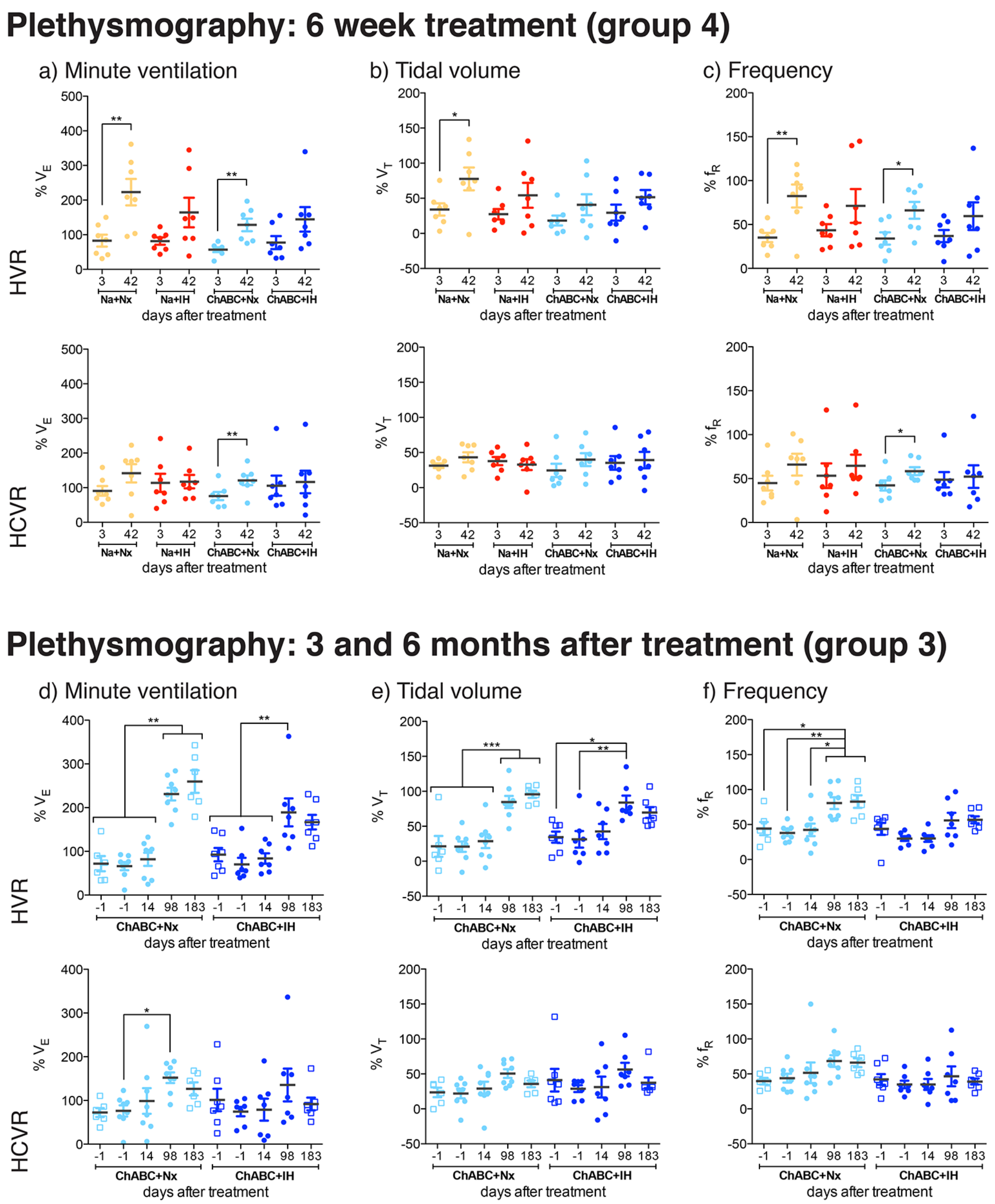

Supplementary Figure 5. The mediatory effects of IH on ventilator response are robust with time. Data shows the ventilatory response (V_E), tidal volume (V_T) and frequency (f_R) following the response to acute hypoxia (10% O_2; HVR) and acute hypercapnia (5% CO_2; HCVR). a-c) During treatment (six weeks following ChABC, 5 weeks IH/Nx), recordings occurred at days three and forty-two. d-f) Recordings occurred three (filled dots) and six months (unfilled squares) after the end of treatment (ChABC and 1 week IH/Nx). For all graph panels treatment groups = saline+air (yellow); saline+IH (red); ChABC+air (light blue); ChABC+IH (blue). * = $p<0.05$, and ** = $p<0.01$(*Bonferroni*) (n=7/8 per group). If no post-hoc result is shown, comparison was not-significant. For all panels: values represent mean±SEM.

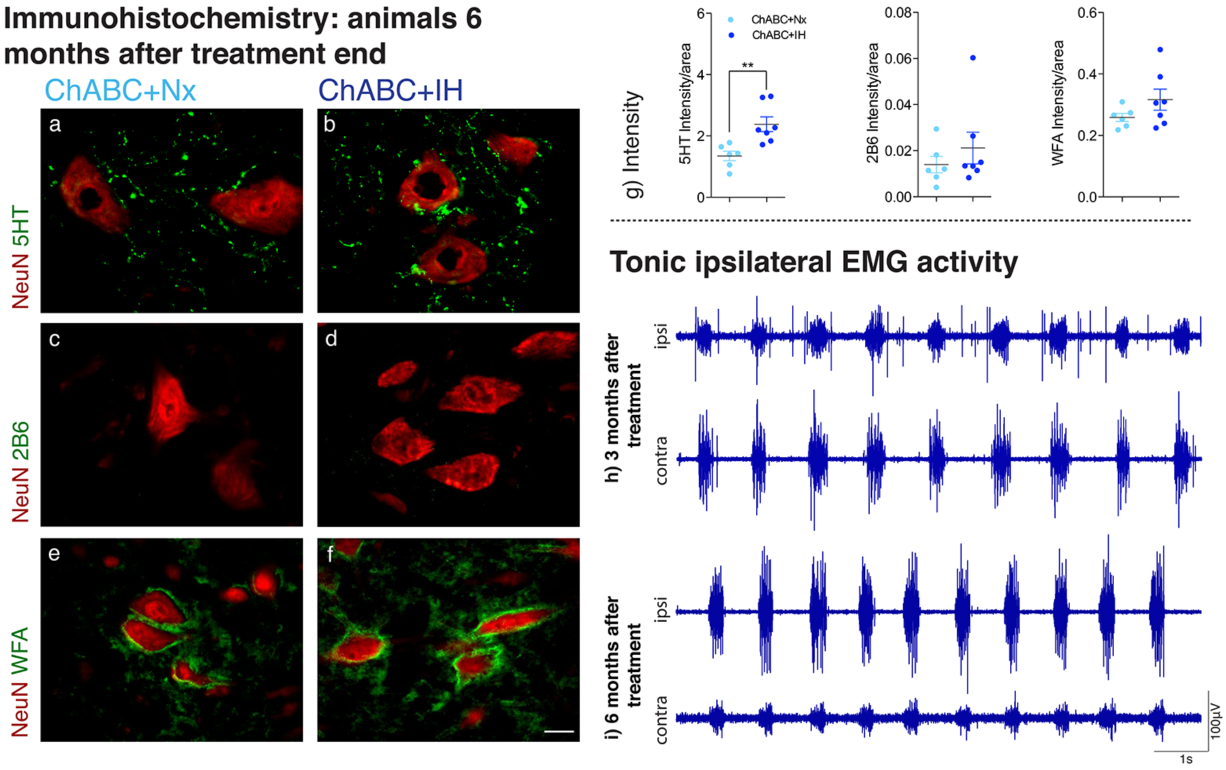

Supplementary Figure 6. Contralateral 5HT is increased six months after treatment while tonic activity ceases. a-g) Immunohistochemistry and intensity readings g) of the putative contralateral PMP six months after ChABC and either air (light blue) or IH conditioning (blue) utilising a-f) NeuN (red) and either (green) a-b) 5HT, c-d) 2B6, or e-f) WFA. ** = $p<0.01$ (*Bonferroni*) (n=6/7 per group). If no post-hoc result is shown, comparison was not-significant. Scale bar = 50µm. Values represent mean±SEM. h-i) Representative diaEMG traces of ChABC+IH treated animals (blue) either h) three or i) six months after treatment end. Ipsilateral tonic motorunit firing was evident at three months (n=2), however, all tonic activity had ceased at six months.

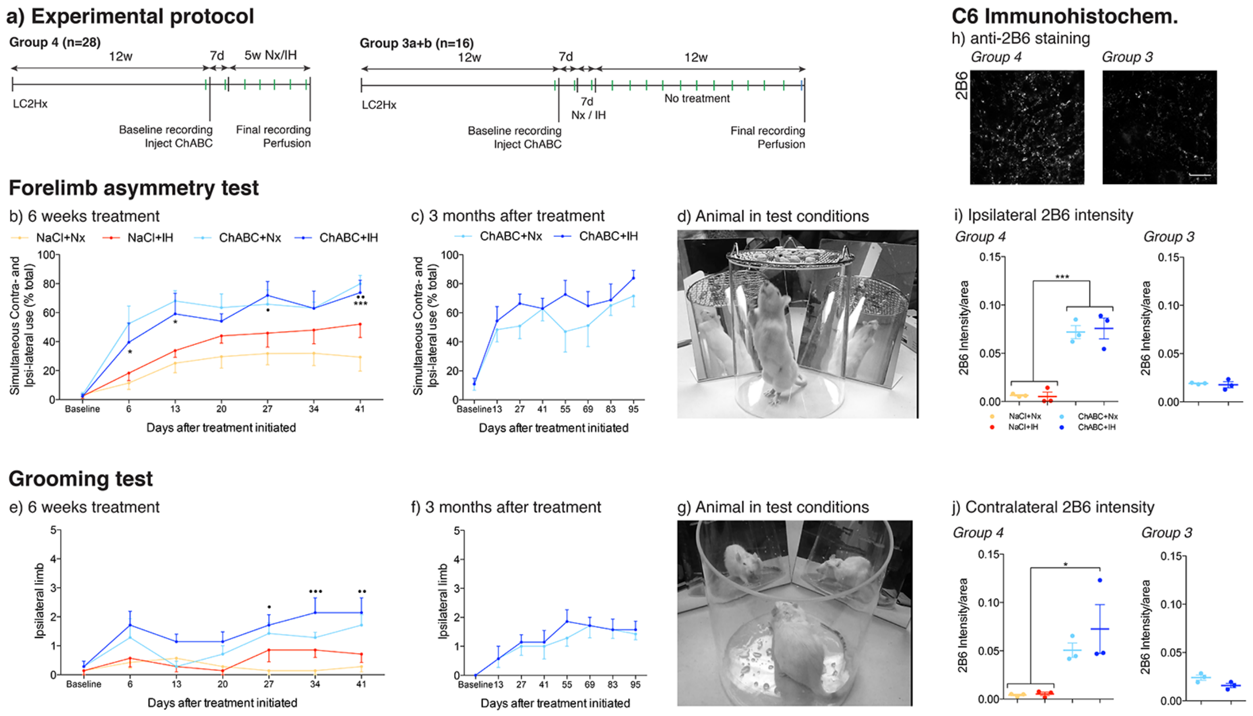

Supplementary Figure 7. ChABC and IH treatment partially increases ipsilateral forelimb motor function. a) Experimental protocol where green ticks mark time points for assessment. b-d) Forelimb asymmetry test with b) 6 weeks of total treatment or c) 3 months after the end of treatment, d) representative animal. e-g) Grooming test with e) 5 weeks of treatment or f) 3 months after the end of treatment, g) representative animal. Figures show raw data and represent mean±SEM. For panels b-g) n=6-8 per group. h-j) Immunohistochemistry (h; ChABC+IH groups shown; scale bar=50µm) and intensity readings (i-j) for 2B6 at the (i) ipsilateral or (j) contralateral intermediate ventral horn at C6 either 6 weeks or 3 months following treatment (n=3 for each group). For graphs: values represent mean±SEM; treatment groups = saline+air (yellow); saline+IH (red); ChABC+air (light blue); ChABC+IH (blue). * = p<0.05, ** = p<0.01, and *** = p<0.001 (*Bonferroni*), where star points of significance = Control vs. ChABC-Nx comparison; and circle points of significance = Control vs. ChABC-IH. If no post-hoc result is shown, comparison was not-significant.

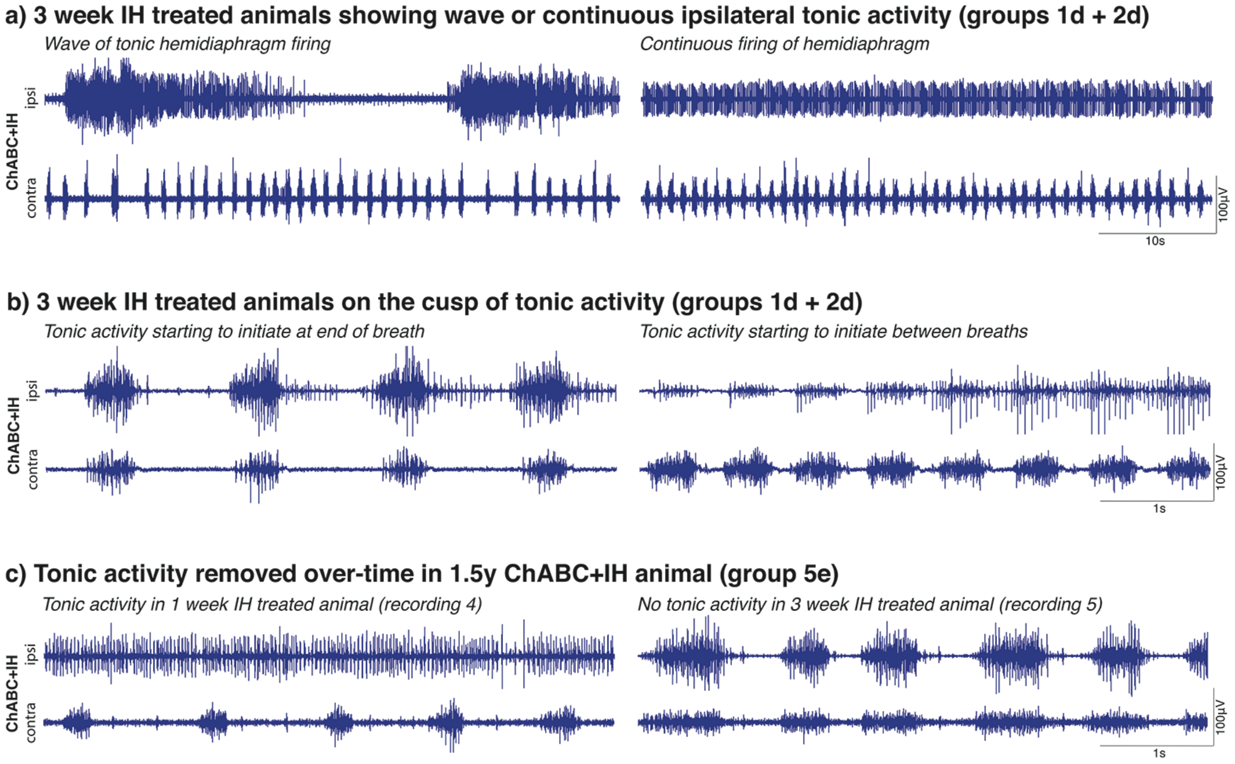

Supplementary Figure 8. Variability in tonic firing of ipsilateral motorneurons. a) The tonic ipsilateral firing occurs either as a repeated wave of firing (associated with a slowing of fR) or is continuously active throughout the recording. b) Representative traces showing two animals with signs of tonic activity. One animal increased firing occurred at the end of a breath, while in another animal it was shown as a short four-second increase in ipsilateral firing. c) Representative diaEMG traces of an animal injured for 1.5-years, displaying tonic ipsilateral firing following one week of conditioning. However, this was resolved with two weeks of conditioning. All panels recorded in ChABC+IH treated animals (blue).

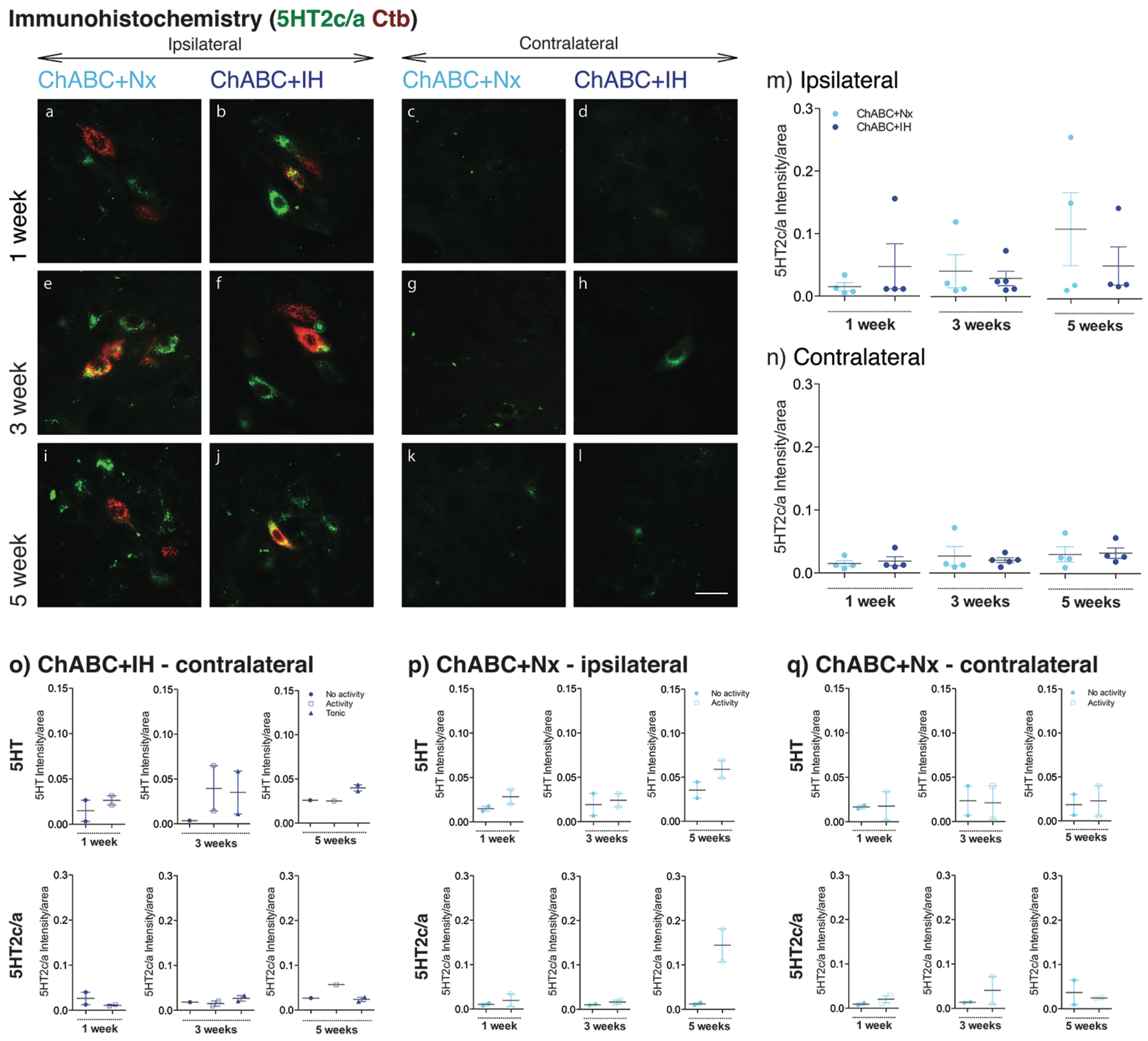

Supplementary Figure 9. ChABC and IH treatment induced tonic ipsilateral hemidiaphragm firing is associated with changes in immunohistochemistry. a-n) Immunohistochemisty and intensity data for the ChABC+Nx and ChABC+IH treated animals with a-d) one, e-h) three, and i-l) five weeks of conditioning stained for 5HT2c/a (green) and Ctb (red). Scale bar = 50µm. The intensity readings for these data are shown for the m) ipsilateral and n) contralateral C4 PMPs (n=5/6 per group). If no post-hoc result is shown, comparison was not-significant. o-q) Intensity data from immunohistochemistry experiments divided into groups based on the activity of the ipsilateral hemidiaphragm: non-responders (circle; n=1/2), normal activity (square; n=1/2), tonic (triangle; n=2). For all graph panels treatment groups = ChABC+air (light blue); ChABC+IH (blue); values represent mean±SEM.

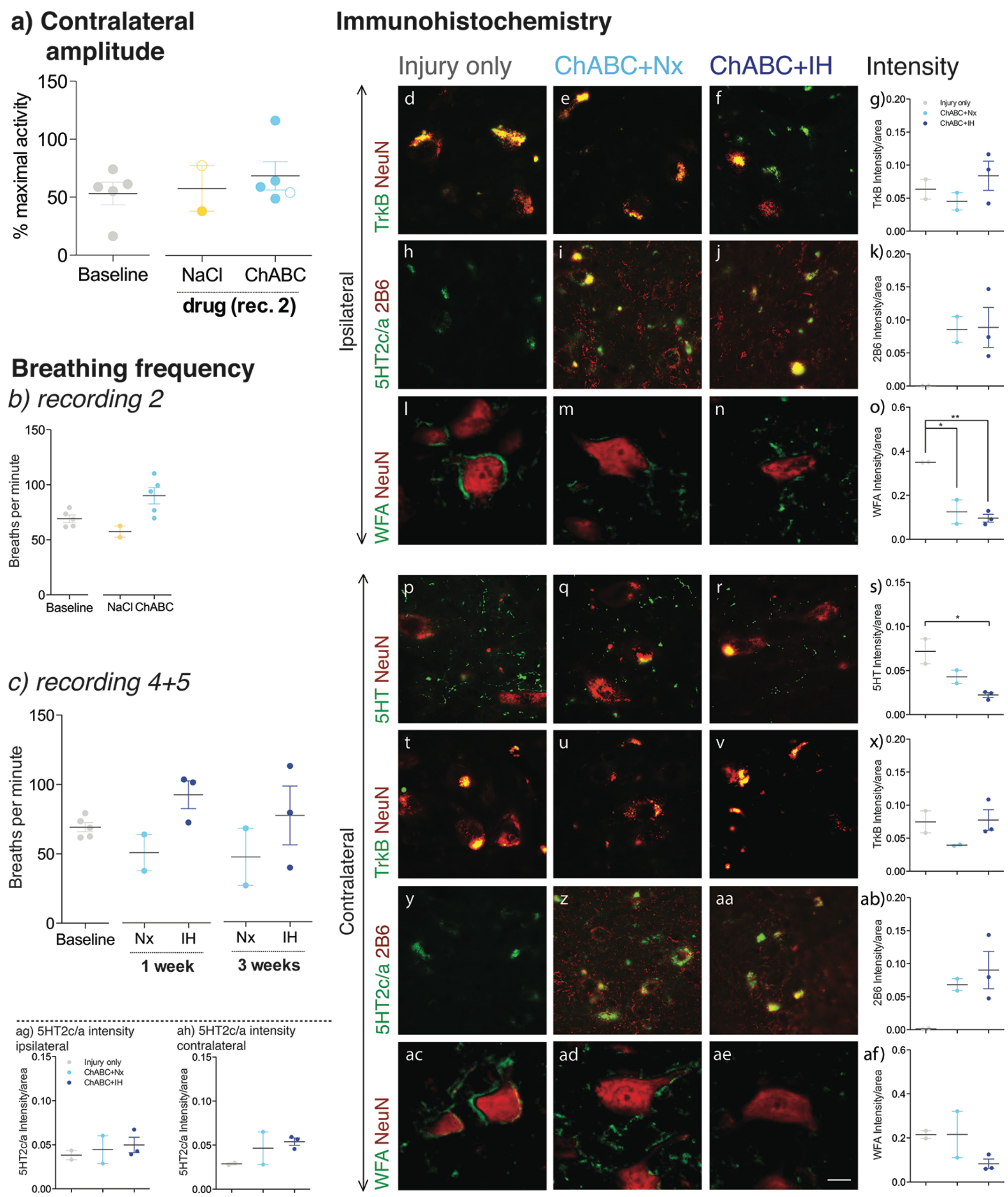

Supplementary Figure 10. PNN breakdown a year and a half following SCI alters respiratory frequency. a) Average amplitude of contralateral diaEMG at baseline (n=5), and following ChABC (n=3) or NaCl (n=2) treatment. b-c) Average number of breaths per minute following b) 1 week and c) 2 weeks of ChABC and air (n=2) or IH (n=3) conditioning compared to baseline (n=5). d-ah) Immunohistochemistry and intensity readings at the d-o and ag) ipsilateral and p-af and ah) contralateral C4 phrenic pool. Stains: d-g and t-x) TrkB (green) and NeuN (red), h-k and y-ab) 5HT2c/a (green) and 2B6 (red), l-o and ac-af) WFA (green) and NeuN (red), and p-s) 5HT (green) and NeuN (red). Scale bar=50µm (n=2/3 per group). For all EMG or graph panels, treatment groups = baseline (grey); saline+air (yellow); ChABC+air (light blue); ChABC+IH (blue). * = $p<0.05$, ** = $p<0.01$, and *** = $p<0.001$ (*Bonferroni*). If no post-hoc result is shown, comparison was not-significant. For all panels: values represent mean±SEM.

Supplementary tables:

Group		Time C2Hx to treatment	Treatment	Weeks of treatment	Time post treatment	# of animals	Reps	Functional measures	Notes
1	a	3 months	NaCl, Nx	4 (NaCl/ChABC = 4, Nx/IH = 3)	N/A	6	3	EMG, IHC, tracing, volumetrics	Additional repeats of groups 1c and 1d occur in group 2
	b		NaCl, IH			6			
	c		ChABC, Nx			6			
	d		ChABC, IH			6			
2	a	3 months	ChABC, Nx	2 (ChABC = 2; Nx/IH=1)	N/A	6	N/A	EMG, IHC, tracing, pharma	Repeats of groups 2e and 2f occur in group 4
	b		ChABC, IH			7			
	c		ChABC, Nx	4 (ChABC = 4, Nx/IH = 3)		6			
	d		ChABC, IH			7			
	e		ChABC, Nx	6 (ChABC = 6; Nx/IH = 5)		6			
	f		ChABC, IH			6			
3	a	3 months	ChABC, Nx	2 (ChABC = 2; Nx/IH=1)	3 months	8	N/A	All: EMG, IHC, pleth. 3a and b: forelimb. 3c and d: NMJ,	
	b		ChABC, IH			8			
	c		ChABC, Nx	2 (ChABC = 2; Nx/IH=1)	6 months	7			
	d		ChABC, IH			7			
4	a	3 months	NaCl, Nx	6 (NaCl/ChABC = 6, Nx/IH = 5)	N/A	7	2	EMG, pharma, IHC, pleth, forelimb, NMJ	
	b		NaCl, IH			7			
	c		ChABC, Nx			7			
	d		ChABC, IH			7			
5	a	1 year	N/A	N/A	N/A	2	N/A	EMG, IHC	Animals from group 5b were treated with ChABC and included in group 5c following their initial NaCl treatment and recording. Animals in group 5c were divided into groups 5d and 5e following 1 week of ChABC treatment.
	b	1.5 years	NaCl	4 (NaCl/ChABC= 1 then ChABC = 4, Nx/IH = 3)		2			
	c		ChABC			5			
	d		ChABC, Nx			2			
	e		ChABC, IH			3			

Supplementary Table 1: Summary table of experimental groups. Animals were allocated to each group based on random number generation. Reps. describes the number of times that a complete experimental group has been repeated. All reps. of experimental groups yielded data statistically non-divergent from that presented within this manuscript. Of the 7 animals in Group 5, 2 were used as controls and tissue collection (group 5a; Figure 6a). The remaining 5 animals were randomly allocated to two groups. Following baseline diaEMG recordings (still showing no spontaneous recovery) group 5b was injected with vehicle into the ipsilateral C4 phrenic motor pool while group 5c had a single injection of ChABC (Figure 6a). 7 days later, the diaEMGs of all animals were recorded. Group 5b then received a ChABC injection and, following a 7-day delay, diaEMGs were recorded. Upon receiving the enzyme, animals were randomly allocated into air or IH treatment groups (groups 5d+e; Figure 6a). Conditioning occurred for 3 weeks with diaEMG recordings occurring serially at the 1 and 3-week time points.

	Amp.	5HT	2B6	TrkB	WFA	GFAP
**Amp.**		0.408 *p*=0.639	0.933 *p*=0.965	0.132 *p*=0.364	0.792 *p*=0.890	0.841 *p*=0.917
**5HT**	0.228 *p*=0.045		0.159 *p*=0.398	0.132 *p*=0.363	0.085 *p*=0.291	0.129 *p*=0.359
**2B6**	0.698 *p*=0.00005	0.524 *p*=0.002		0.174 *p*=0.417	0.006 *p*=0.078	0.431 *p*=0.657
**TrkB**	0.477 *p*=0.003	0.270 *p*=0.39	0.125 *p*=0.178		0.697 *p*=0.835	0.600 *p*=0.775
**WFA**	0.183 *p*=0.098	0.302 *p*=0.027	0.141 *p*=0.151	0.077 *p*=0.298		0.773 *p*=0.879
**GFAP**	0.007 *p*=0.741	0.199 *p*=0.056	0.019 *p*=0.606	0.002 *p*=0.869	0.025 *p*=0.562	

**Ipsilateral:**	0.0 to 0.2	0.2 to 0.4	0.4 to 0.6	0.6 to 0.8	0.8 to 1.0
**Contralateral:**	0.0 to 0.2	0.2 to 0.4	0.4 to 0.6	0.6 to 0.8	0.8 to 1.0

Supplementary Table 2: Correlation matrix between intensity of antibody staining at the PMP and maximal amplitude of the ipsilateral (blue) and contralateral (red) hemidiaphragm showing intensity mapping of the R^2 value (coefficient of determination). Abbreviations: Amp (amplitude), 5HT (serotonin), GFAP (glial fibrillary acidic protein), TrkB (tropomyosin receptor kinase B), WFA (wisteria floribunda agglutinin).

	V_E (mL/g/min)	V_T (mL/g)	f_R (br/min)
	Animals after six weeks ChABC/NaCl and five weeks Nx/IH conditioning		
NaCl + Nx	0.510 ± 0.040	0.00608 ± 0.0005	83.902 ± 0.886
NaCl + IH	0.529 ± 0.038	0.00625 ± 0.00016	84.37 ± 4.751
ChABC + Nx	0.586 ± 0.046	0.00719 ± 0.00033	81.27 ± 4.574
ChABC + IH	0.541 ± 0.034	0.00625 ± 0.00034	86.428 ± 2.201
	Animals three months after treatment end		
ChABC + Nx	0.662 ± 0.082	0.00799 ± 0.00086	82.285 ± 2.643
ChABC + IH	0.592 ± 0.051	0.00653 ± 0.00037	83.87 ± 3.805
	Animals six months after treatment end		
ChABC + Nx	0.584 ± 0.056	0.0071 ± 0.00047	81.466 ± 3.924
ChABC + IH	0.483 ± 0.025	0.00584 ± 0.00039	83.88 ± 4.685

Supplementary Table 3: Baseline ventilatory data for animals the day before treatment application. None of the values are statistically divergent from each other. Animals were split into groups where the terminal recording occurred immediately after the end of six weeks treatment, three months after treatment end, and six months after treatment end. Data shows the ventilatory response (V_E), tidal volume (V_T) and frequency (f_R). Values represent mean±SEM (ANOVA).
