# Supplementary material for: Rapid and robust restoration of breathing long after spinal cord injury
Source: Nat Commun. 2018 Nov 27;9:4843. doi: 10.1038/s41467-018-06937-0 (PMC6258702; doi:10.1038/s41467-018-06937-0)
Supplement: Supplementary file 3 — Description of Additional Supplementary Files [file 41467_2018_6937_MOESM3_ESM.pdf]

Description of Additional Supplementary Files

File Name: Supplementary Movie 1

Description: The inhibitory components in the extracellular matrix (ECM) do not facilitate recovery of ipsilateral diaphragm function following chronic paralysis. Movie shows the ventral diaphragm in an animal treated with saline and air. The paralysed, ipsilateral hemidiaphragm (right of screen) displays no contractile function. This suggests that, without removal of inhibitory ECM components, functional recovery of the ipsilateral hemidiaphragm does not occur. The contralateral hemidiaphragm (left of screen) is functioning normally showing strong rhythmic contractions which, due to the paralysis, pull the ipsilateral hemidiaphragm to the contralateral side of the body. Movie shown at half normal speed.

File Name: Supplementary Movie 2

Description: Plasticity induced by IH conditioning does not recover diaphragm function following chronic paralysis. Movie shows the ventral diaphragm in an animal treated with saline and IH conditioning. No contractile function is shown in the ipsilateral hemidiaphragm (right of screen). This suggests that, despite IH inducing plasticity, without removal of inhibitory ECM components functional recovery of hemidiaphragm activity does not occur. The contralateral hemidiaphragm (left of screen) is functioning normally showing strong rhythmic contractions which, due to the paralysis, pull the ipsilateral hemidiaphragm to the contralateral side of the body. Movie shown at half normal speed.

File Name: Supplementary Movie 3

Description: Digestion of ECM CSPGs, and increasing plasticity, mediates recovery of ipsilateral hemidiaphragm function following chronic paralysis. Movie shows the ventral diaphragm in an animal treated with ChABC and air. Clear recovery of function in ipsilateral diaphragm activity (right of screen) is shown through the strong contraction of muscle. The force of this activity is equal to, and synchronised with, the activity of the contralateral hemidiaphragm (left of screen). This suggests that removal of inhibitory components from the ECM alone is both necessary and sufficient to yield functional diaphragm recovery from paralysis. Movie shown at half normal speed.

File Name: Supplementary Movie 4

Description: The breakdown of CSPGs and further induction of plasticity causes recovery of ipsilateral hemidiaphragm function following chronic paralysis. Movie shows the ventral diaphragm in an animal treated with ChABC and IH conditioning. Recovery of function in ipsilateral diaphragm activity (right of screen) is shown through the strong and synchronised contraction of muscle. The IH conditioning did not statistically increase the amplitude of this muscle activity from that induced by ChABC treatment alone. Movie shown at half normal speed.

File Name: Supplementary Movie 5

Description: A sub-group of animals treated with ChABC and IH condition displayed tonic activity in the ipsilateral hemidiaphragm. In this example, the ipsilateral hemidiaphragm is initially paralysed. However, a wave of strong contractile force is exhibited overtime, demonstrating tonic activity within the muscle which lasts a number of seconds due to increased 5HT caused by modulations to plasticity. diaEMG from this animal is shown in Supplementary Figure 8a. Movie shows the ventral diaphragm and runs at half normal speed.
