# Supplementary material for: Rapid and robust restoration of breathing long after spinal cord injury
Source: Nat Commun. 2018 Nov 27;9:4843. doi: 10.1038/s41467-018-06937-0 (PMC6258702; doi:10.1038/s41467-018-06937-0)
Supplement: Supplementary file 9 — Reporting Summary [file 41467_2018_6937_MOESM9_ESM.pdf]

nature research

Corresponding author(s): Philippa Warren Ph.D.; Warren Alilian Ph.D.; Jerry Silver Ph.D.

Reporting Summary

Nature Research wishes to improve the reproducibility of the work that we publish. This form provides structure for consistency and transparency in reporting. For further information on Nature Research policies, see Authors & Referees and the Editorial Policy Checklist.

Statistical parameters

When statistical analyses are reported, confirm that the following items are present in the relevant location (e.g. figure legend, table legend, main text, or Methods section).

n/a	Confirmed	
☐	☒	The exact sample size (*n*) for each experimental group/condition, given as a discrete number and unit of measurement
☐	☒	An indication of whether measurements were taken from distinct samples or whether the same sample was measured repeatedly
☐	☒	The statistical test(s) used AND whether they are one- or two-sided<br>*Only common tests should be described solely by name; describe more complex techniques in the Methods section.*
☐	☒	A description of all covariates tested
☐	☒	A description of any assumptions or corrections, such as tests of normality and adjustment for multiple comparisons
☐	☒	A full description of the statistics including central tendency (e.g. means) or other basic estimates (e.g. regression coefficient) AND variation (e.g. standard deviation) or associated estimates of uncertainty (e.g. confidence intervals)
☐	☒	For null hypothesis testing, the test statistic (e.g. *F*, *t*, *r*) with confidence intervals, effect sizes, degrees of freedom and *P* value noted<br>*Give P values as exact values whenever suitable.*
☐	☒	For Bayesian analysis, information on the choice of priors and Markov chain Monte Carlo settings
☐	☒	For hierarchical and complex designs, identification of the appropriate level for tests and full reporting of outcomes
☐	☒	Estimates of effect sizes (e.g. Cohen's *d*, Pearson's *r*), indicating how they were calculated
☐	☒	Clearly defined error bars<br>*State explicitly what error bars represent (e.g. SD, SE, CI)*

Our web collection on statistics for biologists may be useful.

Software and code

Policy information about availability of computer code

Data collection	EMG data was collected using CED Spike 2 software; plethysmography using ADinstruments LabChart; Intensity readings for IHC were conducted in ImageJ; movies were recorded in mp4 format and assessed using VPL player; lesion areas were calculated in Adobe Photoshop (cc7)
Data analysis	IBM SPSS software (version 23) or GraphPad Prism (version 5)

For manuscripts utilizing custom algorithms or software that are central to the research but not yet described in published literature, software must be made available to editors/reviewers upon request. We strongly encourage code deposition in a community repository (e.g. GitHub). See the Nature Research guidelines for submitting code & software for further information.

Data

Policy information about availability of data

All manuscripts must include a data availability statement. This statement should provide the following information, where applicable:

- Accession codes, unique identifiers, or web links for publicly available datasets
- A list of figures that have associated raw data
- A description of any restrictions on data availability

The data sets generated and/or analysed during the current study are available from the corresponding authors on reasonable request

Field-specific reporting

Please select the best fit for your research. If you are not sure, read the appropriate sections before making your selection.

☒ Life sciences ☐ Behavioural & social sciences ☐ Ecological, evolutionary & environmental sciences

For a reference copy of the document with all sections, see nature.com/authors/policies/ReportingSummary-flat.pdf

Life sciences study design

All studies must disclose on these points even when the disclosure is negative.

Sample size	Sample size was determined through power analysis calculations which assessed the minimum number of animals required per treatment group. Power analysis was conducted prior to all experiments to ensure n numbers were sufficient to yield reliable data and were within the 88 to 96 percentiles. In instances where power was not sufficient (due to experimental groups being sub-divided based on experimental outcome), analysis was not conducted.
Data exclusions	No data was excluded from analysis.
Replication	All attempts at replication have been successful. Some data sets have been repeated 3 times. This has been reported within Supplementary Figure 1 in the manuscript.
Randomization	Animals were requested in large cohorts (approximately 40+ at a time). Animals were assigned at the point of surgery into one of the experimental groups under investigation through random number generation (produced by a computer). Investigators were blind to which treatment group the animals had been placed in.
Blinding	Investigators were blind to treatment groups during group allocation, data collection, and data analysis.

Reporting for specific materials, systems and methods

Materials & experimental systems

n/a	Involved in the study	
☒	☐	Unique biological materials
☐	☒	Antibodies
☒	☐	Eukaryotic cell lines
☒	☐	Palaeontology
☐	☒	Animals and other organisms
☒	☐	Human research participants

Methods

n/a	Involved in the study	
☒	☐	ChIP-seq
☒	☐	Flow cytometry
☒	☐	MRI-based neuroimaging

Antibodies

Antibodies used

Primary antibodies:
1) Anti-GFAP, Sigma-Aldrich, G3893, clone G-A-5, Lot# 037K4759
2) Anti-NeuN, Merk, MAB377, clone A60, Lot# 2484239.
3) Anti-5HT, Immunostar, 20080; Whole serum from rabbit, Lot#1341001 and 1431001
4) Anti-TrkB, Biosensis, R121-100, clone 23-36, Lot# R121-100304-SH
5) Anti-2B6, Seikagaku, 270432, clone 2-B-6, Lot# A70120
6) Anti-5HT2a/c, abcam, ab37293, Lot# GR217593-1
7) alpha-Bungarotoxin, tetramethylrhodamine conjugate, Thermo Fisher Scientific, T1175, Lot# 1769583
8) Purified Anit-Neurofilament marker, Biolegend, SMI-312, Lot# B212290
9) SV2-s, Developmental Studies Hydridoma Bank, SV2, Lot# 1/28/16-45Ag/mL

Secondary antibodies, and streptavidin:
1) Flourescin goat-anti-mouse IgG1, Thermo Fisher Scientific, A10530, Lot# 1818304
2) Goat anti Rabbit Alexa Fluor 488, Thermo Fisher Scientific, A11008
3) Goat anti Rabbit Alexa Fluor 594, Thermo Fisher Scientific, A11012
4) Goat anti Mouse IgG Alexa Fluor 594, Thermo Fisher Scientific, A11005
5) Goat anti Mouse IgG Alexa Fluor 488, Thermo Fisher Scientific, A11001
6) Goat anti Mouse IgM Alexa Fluor 488, Thermo Fisher Scientific, A21042

7) Goat anti Mouse IgM Alexa Fluor 594, Thermo Fisher Scientific, A21044

Validation

1) Anti-GFAP, Sigma-Aldrich, G3893, clone G-A-5, Lot# 037K4759
Validation: company website states antibody should be used for immunohistochemistry on human, pig and rat (http://www.sigmaaldrich.com/catalog/product/sigma/g3893?lang=en&region=GB). It has been used in numerous citations (300+) including 1) Dusonchet et al. (2011) J. Neurosci. 31(3), 907-912. 2) Alilain et al. (2011) Nature, 475(7355) 196-200. 3) Colangelo et al. (2008) J. Neurosci. 28(11), 2698-2709. Prior to use the antibody was tested in positive and negative control samples of rat tissue known either to express or not express the desired epitope. The resultant images were compared to known test samples to ensure the correct labelling.

2) Anti-NeuN, Merk, MAB377, clone A60, Lot# 2484239.
Validation: company website states antibody should be used for immunohistochemistry in multiple species including rat (http://www.merckmillipore.com/GB/en/product/Anti-NeuN-Antibody%2C-clone-A60,MM_NF-MAB377?ReferrerURL=https%3A%2F%2Fwww.google.co.uk%2F&bd=1). It has been used in numerous citations (2000+) including 1) Tsutsui-Kimura et al. (2014) Int. J. Neuropsychopharma, 18(51), 1-14. 2) Vicuna et al. (2015) Nat. Med., 21(5), 518-523. Prior to use the antibody was tested in positive and negative control samples of rat tissue known either to express or not express the desired epitope. The resultant images were compared to known test samples to ensure the correct labelling.

3) Anti-5HT, Immunostar, 20080; Whole serum from rabbit, Lot#1341001 and 1431001
Validation: company website states antibody should be used for immunohistochemistry in numerous species including the rat (http://www.immunostar.com/shop/antibody-catalog/5-ht-serotonin-rabbit-antibody/). It has been used in numerous (1000+) citations including 1) Alilain et al. (2011) Nature, 475(7355) 196-200. 2) De Paul et al. (2015) PLoS ONE, 10(10), e0139335. 3) Ishikawa et al. (2015) Neurosci. Letters, 593, 13-18. Prior to use the antibody was tested in positive and negative control samples of rat tissue known either to express or not express the desired epitope. The resultant images were compared to known test samples to ensure the correct labelling.

4) Anti-TrkB, Biosensis, R121-100, clone 23-36, Lot# R121-100304-SH
Validation: company website states antibody should be used for immunohistochemistry in numerous species including the rat (http://www.biosensis.com/rabbit-antibody-tyrosine-kinase-receptor-trkb-whole-serum-p-198.html). It has been used in numerous citations including
Prior to use the antibody was tested in positive and negative control samples of rat tissue known either to express or not express the desired epitope. The resultant images were compared to known test samples to ensure the correct labelling.

5) Anti-2B6, Seikagaku, 270432, clone 2-B-6, Lot# A70120
Validation: company website states antibody should be used for immunohistochemistry (http://www.amsbio.com/productpage.aspx?code=270432-1&cty=UNITED%20KINGDOM&cur=GBP). It has been used in numerous citations including 1) Alilain et al. (2011) Nature, 475(7355) 196-200. 2) Couchman et al. (1984) Nature, 307(5952), 650-652. 3) Kim et al. (2006) J. Comp. Neurol., 497(2) 182-198. Prior to use the antibody was tested in positive and negative control samples of rat tissue known either to express or not express the desired epitope. The resultant images were compared to known test samples to ensure the correct labelling.

6) Anti-5HT2a/c, abcam, ab37293, Lot# GR217593-1
Validation: company website states antibody should be used for immunohistochemistry in two species including the rat (http://www.abcam.com/5ht2c-receptor-antibody-ab37293.html). It has been used in numerous citations including 1) Murray et al. (2010) Nat. Med., 16, 694-700. Prior to use the antibody was tested in positive and negative control samples of rat tissue known either to express or not express the desired epitope. The resultant images were compared to known test samples to ensure the correct labelling.

7) alpha-Bungarotoxin, tetramethylrhodamine conjugate, Thermo Fisher Scientific, T1175, Lot# 1769583
Validation: company website states antibody should be used for immunohistochemistry (https://assets.thermofisher.com/TFS-Assets/LSG/manuals/mp01175.pdf). It has been used in citations for assessment of neuromuscular junctions including 1) Martin, et al. (2015) J Vis Exp e52605. Prior to use the antibody was tested in positive and negative control samples of rat tissue known either to express or not express the desired epitope. The resultant images were compared to known test samples to ensure the correct labelling.

8) Purified Anit-Neurofilament marker, Biolegend, SMI-312, Lot# B212290
Validation: company website states antibody should be used for immunohistochemistry (https://www.biolegend.com/en-us/products/purified-anti-neurofilament-marker-pan-axonal-cocktail-12811). It has been used in citations for assessment of neuromuscular junctions including 1) Martin, et al. (2015) J Vis Exp e52605. Prior to use the antibody was tested in positive and negative control samples of rat tissue known either to express or not express the desired epitope. The resultant images were compared to known test samples to ensure the correct labelling.

9) SV2-s, Developmental Studies Hydridoma Bank, SV2, Lot# 1/28/16-45Ag/mL
Validation: company website states antibody can be used for immunohistochemistry (http://dshb.biology.uiowa.edu/synaptic-vesicles). It has been used in citations for assessment of neuromuscular junctions including 1) Martin, et al. (2015) J Vis Exp e52605. Prior to use the antibody was tested in positive and negative control samples of rat tissue known either to express or not express the desired epitope. The resultant images were compared to known test samples to ensure the correct labelling.

10) Flourescin goat-anti-mouse IgG1, Thermo Fisher Scientific, A10530, Lot# 1818304
Validation: company website states antibody should be used for immunohistochemistry (https://www.thermofisher.com/antibody/product/Goat-anti-Mouse-IgG1-Cross-Adsorbed-Secondary-Antibody-Polyclonal/A10530). It has been used in citations for assessment of neuromuscular junctions including 1) Martin, et al. (2015) J Vis Exp e52605. Prior to use the antibody was tested in positive and negative control samples of rat tissue known either to express or not express the desired epitope. The resultant images were compared to known test samples to ensure the correct labelling.

Animals and other organisms

Policy information about studies involving animals; ARRIVE guidelines recommended for reporting animal research

Laboratory animals	Adult female Sprague Dawley rats (200-250g) were used at either 3 months old at the point of initial injury (any animal that was treated 3 months after the spinal injury) or a retired breeder (~6-9 months old; animals that were treated 1.5 years after the spinal injury).
Wild animals	No wild animal was used in the study
Field-collected samples	No field-collected samples were used in this study
